# The sine transform is the *sine qua non* of the pulmonary and systemic pressure relationship

**DOI:** 10.3389/fcvm.2023.1120330

**Published:** 2023-05-26

**Authors:** Mark Doyle, Geetha Rayarao, Robert W. W. Biederman

**Affiliations:** Department Cardiology, Cardiovascular MRI, Cardiovascular Institute, Allegheny Health Network, Pittsburgh, PA, United States

**Keywords:** pulmonary hypertension, pulmonary arterial hypertension, mathematical model, physiology, ventricular interdependance

## Abstract

Assessment of therapeutic interventions in patients with pulmonary arterial hypertension (PAH) suffers from several commonly encountered limitations: (1) patient studies are often too small and short-term to provide definitive conclusions, (2) there is a lack of a universal set of metrics to adequately assess therapy and (3) while clinical treatments focus on management of symptoms, there remain many cases of early loss of life in a seemingly arbitrary distribution. Here we provide a unified approach to assess right and left pressure relationships in PAH and pulmonary hypertension (PH) patients by developing linear models informed by the observation of Suga and Sugawa that pressure generation in the ventricle (right or left) approximately follows a single lobe of a sinusoid. We sought to identify a set of cardiovascular variables that either linearly or via a sine transformation related to systolic pulmonary arterial pressure (PAPs) and systemic systolic blood pressure (SBP). Importantly, both right and left cardiovascular variables are included in each linear model. Using non-invasively obtained cardiovascular magnetic resonance (CMR) image metrics the approach was successfully applied to model PAPs in PAH patients with an *r*^2^ of 0.89 (*p* < 0.05) and SBP with an *r*^2^ of 0.74 (*p* < 0.05). Further, the approach clarified the relationships that exist between PAPs and SBP separately for PAH and PH patients, and these relationships were used to distinguish PAH vs. PH patients with good accuracy (68%, *p* < 0.05). An important feature of the linear models is that they demonstrate that right and left ventricular conditions interact to generate PAPs and SBP in PAH patients, even in the absence of left-sided disease. The models predicted a theoretical right ventricular pulsatile reserve that in PAH patients was shown to be predictive of the 6 min walk distance (*r*^2^ = 0.45, *p* < 0.05). The linear models indicate a physically plausible mode of interaction between right and left ventricles and provides a means of assessing right and left cardiac status as they relate to PAPs and SBP. The linear models have potential to allow assessment of the detailed physiologic effects of therapy in PAH and PH patients and may thus permit cross-over of knowledge between PH and PAH clinical trials.

## 1. Introduction

We sought to develop linear models of pulmonary and systemic pressure to provide a universal set of metrics to address key paradoxes that exist in the study of PAH and PH patients (1). Firstly, PAH, a rare, untreatable and progressive disease has dominated the interests of basic, translation and clinical scientist and the pharmaceutical industry. From the time of detection there is a 5–7 year half mortality burden (2). Conversely, PH is common, often secondary to left heart disease, and remains largely undetected until severe and irreversible damage is sustained, leading to a very poor prognosis once discovered (3–6). Secondly, the PAH population remains so small that traditional clinical trials often fail to find any statistically significant impact of treatment, despite the common observation that some patients demonstrate clinically relevant improvement (7). A recent meta-analysis of the use of time to clinical worsening as an end-point in PAH trials noted that while it may lead to shorter and smaller trials, time to clinical worsening cannot be considered as valid surrogates for mortality in PAH trials (8) and further, that there is no widely-agreed upon definition of this metric (9). Additionally, the distinction between PAH and PH can be clinically challenging, with each cohort presenting with similar symptoms (10). This difficulty is further confounded in patients with a combination of pre and post-capillary causes of PH (11). Many of the above difficulties in detection and characterization relate to a lack of access to key variables that adequately assess PA pressure and cardiac status (12–16). In part this is due to the wide variety of hemodynamic indices and concepts employed to characterize PAH and in part due to the difficulty in obtaining key measures of cardiac status such as cardiac outflow reserve. While right ventricular outflow reserve has been shown to be a good prognostic indicator, since it involves a time-consuming and potentially hazardous rest and stress examination, it is rarely performed and even more rarely performed in an ongoing manner during routine clinical assessment (17).

In developing a unified pair of linear models capable of characterizing and assessing both PAH and PH patients we consider the disease state commonalties as well as differences. The widely perceived major difference involves the role of the left ventricle (LV). While PH is predominantly the result of left-sided disease, PAH is, by strict World Health Organization (WHO) classification, not related to left-sided disease (18). In PAH this often leads to neglect of left-sided conditions and considerations (19). However, ventricular interdependence is inevitable due to (A) the non-compliant pericardial sac constraining the total volume of the combined left and right ventricles, (B) the left and right ventricles being connected in series and thus constrained to generate the same stroke volume, and (C) that both ventricles share a common septum which directly transmits pressure from one to the other (20). These conditions inform us of the mechanisms by which the ventricles interact and indicate that that both ventricles contribute to right and left pressure generation. However, they do not provide any quantitative means of assessing what the pressure conditions are and how in detail they relate. To develop quantitative linear models of pressure we hypothesized that various key cardiac variables adequately define the system’s “state variables”. State variables are a set of physical conditions such as ejection fraction (EF) and end systolic volume (ESV) that describe the state of the dynamic cardiovascular system (21). In this representation of the cardiovascular system, the heart generates pulmonary and systemic blood pressures via time-evolution of its state variables. Suga and Sugawa observed that during isovolumic contraction and relaxation, pressure generation can be modeled by a sinusoidal waveform (22, 23). Thus, we sought to identify the cardiac state variables by applying a sinusoidal transform to candidate variables to model blood pressure. The linear models presented here were successfully applied to PAH patients, clearly showing the mode and magnitude of linkage between right and left-ventricular pressure generation. Further the linear models identified key differences in the manner of pressure generation between PAH and PH patients despite a similar range of PAPs. Finally, the models naturally suggested the concept of RV contractile reserve which was shown to correlate with the 6 min walk distance (6MWD). The ultimate value of the linear models is that they provide a set of non-invasively obtainable quantitative metrics that can be used to assess both pulmonary and systemic pressure conditions. In particular, in the context of clinical trials that target structural remodeling directly, there is a shortage of suitable approaches to measure markers of biology that drive cardiovascular change (24). Even positive PAH trials suffer from a low rate of reproducibility (as low as 21%) in part due to the adequacy of the markers of benefit (25). Consider that improvement in the 6MWD only poorly correlates with survival benefit (26) and more recently, dependence on time to clinical worsening requires larger trials of longer duration (27). While 4D flow CMR can assess pulmonary arterial pressure via direct interrogation of the flow field, it lacks an assessment of cardiac metrics that correspond to pressure conditions (28). Further, echocardiography is most commonly used to non-invasively assess pulmonary artery pressure but primarily relies on assessment of leakage of the tricuspid valve, which may not be present in each case (29). Thus, our intent in developing linear models of pulmonary and systemic pressure was to identify the anatomic cardiac variables that could be directly measured to assess response to therapy.

## 2. Materials and methods

### 2.1. Study populations

#### 2.1.1. Exclusive PAH cohort (complexa trial patients)

Data were collected from patients with clinically diagnosed pulmonary arterial hypertension (*n* = 51) who were enrolled in the Complexa clinical trial (30). In brief, this trial was a multicenter double-blinded, placebo-controlled study to evaluate the safety, efficacy, and pharmacokinetics of the study drug, CXA-10 [10-nitrooctadec-9(*E*)-enoic acid] which is an endogenous compound. Subjects 18 to 80 years of age (target *n* = 96) with PAH were randomly assigned to 75 mg, 150 mg of CXA-10 or stable background therapy. This phase II trial was terminated early due to lack of efficacy (with no safety concerns). Only baseline data are reported here prior to administration of the study drug. The study was performed at multiple sites from August 1, 2018 to August 5, 2020 (see Supplementary Material Table S1). Approximately 4% of data was missing at random.

In the Complexa trial, all pulmonary hypertension patients with normal pulmonary capillary wedge pressure (<15 mm Hg) or normal left ventricular end diastolic pressure (<10 mm Hg) were diagnosed to have PAH. Upon enrollment, demographic data were collected along with 6MWD data, right heart catheterization (RHC) pressure data, and cardiovascular magnetic resonance (CMR) imaging assessment of the left and right heart. The CMR data was analyzed at our core lab. The key CMR image acquisitions were (1) a multi-stack short axis cine set of images covering the left and right ventricles and used to obtain cardiac chamber volumes, and (2) phase velocity quantitative flow scans positioned through the ascending aorta and main pulmonary artery. All baseline CMR, RHC and 6MWD measurements were performed within a thirty day period of each other. Assignment to WHO functional class was performed at each site using standard of care clinical assessments (functional class data were available in 49 patients, 94%). Baseline demographic and test measurements are provided in Table 1.

**Table 1:** Baseline demographics and measurement of exclusively PAH patients.

Variable	Full population (52)	WHO functional class II (49)	WHO functional class III (17)	*p* range
**General demographics**				
Age (years)	50.37 (SD 12.19)	49.95 (SD 12.84)	50.39 (SD 9.99)	0.91
Height (cm)	162.14 (SD 6.97)	162.4 (SD 7)	161.12 (SD 6.79)	0.55
Weight (kg)	94.6 (SD 29.71)	91.95 (SD 29.84)	100.82 (SD 29.92)	0.33
Male	6 (12.24%)	4 (12.9%)	2 (14.29%)	0.9
**Ethnicity**				
White	36 (73.47%)	24 (77.42%)	9 (64.29%)	0.36
Black	4 (8.16%)	1 (3.23%)	2 (14.29%)	0.17
Hispanic	8 (16.33%)	4 (12.9%)	3 (21.43%)	0.47
Other	9 (18.37%)	6 (19.35%)	3 (21.43%)	0.87
**Substances**				
Alcohol: current user	14 (29.17%)	10 (32.26%)	3 (21.43%)	0.46
Alcohol: former user	8 (16.67%)	7 (22.58%)	1 (7.14%)	0.21
Tobacco use	12 (25%)	9 (29.03%)	2 (14.29%)	0.29
Drug use				0.3
**Respiratory**				
Respiratory rate (breaths/min)	17.38 (SD 2.51)	16.81 (SD 2.09)	18.53 (SD 2.96)	<0.05
Forced expiratory volume in 1 s (%)	79.39 (SD 13.91)	80.1 (SD 12.26)	74.79 (SD 15.57)	0.22
Total lung capacity (%)	89.69 (SD 11.86)	90.4 (SD 11.22)	87.79 (SD 14.57)	0.52
**PAH type**				
Idiopathic	35 (71.43%)	24 (77.42%)	10 (71.43%)	0.67
Connective tissue	9 (18.37%)	3 (9.68%)	3 (21.43%)	0.28
Toxin/drug	2 (4.08%)	1 (3.23%)	1 (7.14%)	0.56
**6 min walk test**				
Oxygen saturation pre (%)	96 (94.25–98.75)	96 (94.5–98)	97 (94.25–98)	0.81
Oxygen saturation post (%)	93.5 (88–96.75)	92 (87–96)	94 (91.25–96.75)	0.78
Heart rate pre (beats/min)	81.28 (SD 11.9)	80.65 (SD 12.25)	81.86 (SD 12.66)	0.76
Heart rate post (beats/min)	112.96 (SD 35.75)	121.6 (SD 17.25)	109.36 (SD 19.37)	<0.05
Distance (m)	402.76 (SD 84.75)	425.13 (SD 74.31)	364.14 (SD 101.84)	<0.05
**Electrocardiographic**				
Mean heart rate (beats/min)	74.97 (SD 11.69)	73.73 (SD 11.72)	78.14 (SD 12.82)	0.26
PR interval (ms)	175.76 (SD 34.64)	169.9 (SD 29.77)	190.05 (SD 44.52)	0.08
QRS duration (ms)	90.53 (SD 11.5)	90.4 (SD 11.28)	93.45 (SD 11.46)	0.41
QT interval (ms)	410.86 (SD 30.25)	412.37 (SD 31.18)	403.52 (SD 30.7)	0.38
QTcF interval (ms)	440.8 (SD 22.64)	439.34 (SD 19.81)	438.95 (SD 29.32)	0.96
RR interval (ms)	799.27 (SD 144.38)	801.92 (SD 155.47)	793.56 (SD 143.24)	0.87
**Right heart catheterization**				
Cardiac output (L/min)	5 (4.15–5.61)	4.82 (4.03–5.39)	5.7 (4.9–6.3)	<0.05
Heart rate (beats/min)	76.31 (SD 10.46)	75.39 (SD 11.06)	78.75 (SD 8.84)	0.36
Mixed venous oxygen saturation (%)	68.12 (SD 13.71)	69.63 (SD 10.76)	70.13 (SD 8.37)	0.89
Systemic oxygen saturation by pulse oximeter (%)	92.07 (SD 14.07)	93.46 (SD 3.99)	94.17 (SD 3.33)	0.6
Mean right atrial pressure (mm Hg)	7.13 (SD 4.01)	6.98 (SD 3.44)	9.15 (SD 5.06)	0.11
Pulmonary artery diastolic pressure (mm Hg)	29 (SD 8.99)	29.9 (SD 9.97)	31 (SD 6.87)	0.72
Pulmonary artery systolic pressure (mm Hg)	76.29 (SD 18.42)	78.37 (SD 19.22)	79.54 (SD 17.27)	0.85
Pulmonary artery wedge pressure (mm Hg)	9.06 (SD 3.02)	9.21 (SD 3)	9.64 (SD 2.66)	0.69
Right ventricular end-diastolic pressure (mm Hg)	7.8 (SD 6.53)	7.17 (SD 5.93)	11.46 (SD 7.38)	0.05
Right ventricular systolic pressure (mm Hg)	73.65 (SD 22.99)	76.57 (SD 22.96)	79.15 (SD 17.11)	0.72
**Systemic**				
Systolic blood pressure (mm Hg)	114.98 (SD 14.86)	116 (SD 15.95)	112.5 (SD 14.24)	0.52
Diastolic blood pressure (mm Hg)	70.13 (SD 9.8)	70.63 (SD 11.4)	68.42 (SD 5.87)	0.53
**Valvular disease**				
Mitral	4 (8.33%)	2 (6.9%)	2 (13.33%)	0.48
Tricuspid	17 (35.42%)	11 (37.93%)	6 (40%)	0.89
Aortic	0 (0%)	0 (0%)	0 (0%)	
Pulmonary	6 (12.5%)	2 (6.9%)	4 (26.67%)	0.07
None	30 (62.5%)	18 (62.07%)	8 (53.33%)	0.58
**CMR variables**				
Aortic flow (ml)	65.83 (SD 17.59)	62.91 (SD 14.13)	71.65 (SD 23.8)	0.13
MPA flow (ml)	63.34 (SD 19.04)	64.28 (SD 18.9)	62.97 (SD 22.06)	0.84
LV mass (g)	116.11 (SD 25.16)	109.24 (SD 23.73)	126.36 (SD 24.68)	<0.05
LV stroke volume (ml)	64.03 (SD 14.66)	61.56 (SD 12.86)	67.45 (SD 18.87)	0.23
LV end diastolic volume (ml)	96.67 (SD 20.34)	92.92 (SD 18.18)	101.73 (SD 23.39)	0.17
LVEF (%)	66.52 (SD 8.41)	66.44 (SD 6.8)	66.23 (SD 9.86)	0.94
LV end systolic volume (ml)	32.64 (SD 11.56)	31.36 (SD 9.38)	34.28 (SD 12.67)	0.39
RV cardiac output (L/min)	4.68 (SD 1.29)	4.55 (SD 1.22)	5.08 (SD 1.45)	0.21
RV mass (g)	105.84 (SD 34.07)	99.43 (SD 33.72)	115.44 (SD 30.04)	0.13
RV stroke volume (ml)	64.68 (SD 16.68)	63.2 (SD 16.74)	67.95 (SD 18.14)	0.39
RV end diastolic volume (ml)	147.58 (SD 38.27)	144.12 (SD 38.22)	151.51 (SD 34.58)	0.53
RVEF (%)	45.37 (SD 11.36)	45.26 (SD 10.64)	45.99 (SD 11.95)	0.84
RV end systolic volume (ml)	82.89 (SD 34.97)	80.92 (SD 34.71)	83.56 (SD 32.73)	0.81
BSA (m^2^)	1.83 (SD 0.56)	1.91 (SD 0.56)	1.68 (SD 0.55)	0.21
LV mass index (g/m^2^)	69.64 (SD 28.29)	62.68 (SD 24.66)	83.09 (SD 30.77)	<0.05
LV stroke volume index (ml/m^2^)	38.23 (SD 14.65)	35.3 (SD 12.36)	43.89 (SD 17.36)	0.06
LV end diastolic volume index (ml/m^2^)	57.6 (SD 20.84)	52.96 (SD 17.13)	66.58 (SD 24.83)	<0.05
LV end systolic volume index (ml/m^2^)	19.37 (SD 8.56)	17.65 (SD 6.35)	22.7 (SD 11.24)	0.06
RV cardiac output index (L/m/m^2^)	2.86 (SD 1.17)	2.59 (SD 0.92)	3.37 (SD 1.44)	<0.05
RV mass index (g/m^2^)	62.38 (SD 26.07)	55.03 (SD 20.04)	76.59 (SD 30.92)	<0.01
RV stroke volume index (ml/m^2^)	39.02 (SD 15.04)	36.31 (SD 13.58)	44.27 (SD 16.77)	0.1
RV end diastolic volume index (ml/m^2^)	87.62 (SD 32.5)	80.64 (SD 25.21)	101.1 (SD 40.95)	<0.05
RV end systolic volume index (ml/m^2^)	48.59 (SD 23.53)	44.33 (SD 17.34)	56.83 (SD 31.45)	0.1
LV EDV/LV mass index (ml/g/m^2^)	0.85 (SD 0.18)	0.87 (SD 0.2)	0.82 (SD 0.17)	0.35
LV impedance match	8.51 (SD 3.59)	8.2 (SD 3.56)	9.15 (SD 3.71)	0.44
RV impedance match	3.75 (SD 1.94)	3.88 (SD 1.92)	3.48 (SD 2.02)	0.54

#### 2.1.2. Suspected PH/PAH cohort

In addition to the Complexa trial patients we obtained data from a retrospective cohort presenting at our CMR facility. Other than selecting patients with sufficient CMR metrics to model pressure, no exclusion criteria were applied. The key CMR acquisition protocol was identical to that used for the Complexa trial patients. Data were collected from 49 consecutive patients referred to our CMR laboratory from 2011 to 2015 who had a suspicion of PH (34, 70%) or PAH (15, 30%). While SBP was measured in all patients, contemporaneous measures of PAPs were not available, while estimates of PAPs were available in 17 (34%) with 10/17 (59%) obtained by echocardiographic criteria involving measurement of the tricuspid regurgitation jet (33). Due to the sparsity and the low quality of the PAPs estimates these pressure data were not used to generate the linear model of PAPs. The purpose of including this patient cohort was to (1) extend the number of patients contributing to the SBP model and (2) to apply the linear models to identify key differences in pressure distribution between PAH and PH patients. Demographic and key test measures of this cohort are provided in Table 2.

**Table 2:** Baseline demographics and measurement of suspected PH/PAH patients.

Variable	Full population (34)	PAH (15)	PH (35)	*p* range
**General demographics**				
Age (years)	52.35 (SD 17.5)	60.93 (SD 14.08)	48.56 (SD 17.7)	<0.05
Height (cm)	167.28 (SD 12.13)	166.62 (SD 9.78)	167.57 (SD 13.15)	0.80
Weight (kg)	82.84 (SD 25.57)	93.11 (SD 25.37)	78.31 (SD 24.68)	0.06
Male	19 (38.78%)	4 (26.67%)	15 (44.12%)	0.25
**Blood pressures**				
Estimated pulmonary artery systolic pressure (mm Hg)	50.12 (SD 23.47)	55.86 (SD 28.71)	46.1 (SD 19.66)	0.42
Measured systemic systolic blood pressure (mm Hg)	133.84 (SD 16.41)	137.91 (SD 17.91)	132.05 (SD 15.65)	0.25
**Key CMR variables**				
LV mass (g)	99.45 (SD 36.78)	98.73 (SD 26.63)	99.76 (SD 40.82)	0.93
RV mass (g)	46.89 (SD 15.39)	49.37 (SD 13.81)	45.79 (SD 16.11)	0.46
RVEF (%)	54.06 (SD 9.94)	51.57 (SD 10.08)	55.15 (SD 9.83)	0.25
LVEF (%)	56.2 (SD 7.93)	58.11 (SD 5.4)	55.35 (SD 8.76)	0.26
LV impedance match	8.07 (SD 3.65)	7.53 (SD 3.49)	8.31 (SD 3.75)	0.49
RV impedance match	8.1 (SD 3.68)	6.87 (SD 3.9)	8.65 (SD 3.5)	0.12
LV end systolic volume (ml)	65.61 (SD 27.22)	66.67 (SD 18.71)	65.15 (SD 30.48)	0.86
RV end systolic volume (ml)	91.04 (SD 43.66)	103.53 (SD 43.16)	85.53 (SD 43.36)	0.19
BSA (m^2^)	1.94 (SD 0.34)	2.06 (SD 0.32)	1.89 (SD 0.34)	0.12
LV mass index (g/m^2^)	4.39 (SD 1.95)	4.33 (SD 1.67)	4.42 (SD 2.08)	0.88
RV mass index (g/m^2^)	24.34 (SD 7.93)	24.3 (SD 7.21)	24.36 (SD 8.33)	0.98
LV end systolic volume index (ml/m^2^)	16.59 (SD 7.15)	16.76 (SD 6.21)	16.51 (SD 7.61)	0.91
RV end systolic volume index (ml/m^2^)	5.95 (SD 2.69)	6.43 (SD 2.36)	5.74 (SD 2.83)	0.41

### 2.2. CMR variables

Our hypothesis is that a limited number of key cardiac measures define the state variables of the pulmonary and systemic pressure systems. We sought to identify cardiac metrics obtained non-invasively via CMR imaging. The multi-slice short axis data sets, time resolved through the cardiac cycle and covering the left and right ventricles from base to apex, allowed standard ventricular metrics to be obtained separately for the left and right ventricles, including: EF, ESV and ventricular mass. In addition to the standard CMR functional examination (32), separate phase velocity mapping scans were obtained to quantify blood flow velocity through the ascending aorta just above the sinus and through the main pulmonary artery. This data, obtained at the interface of each ventricle to the vasculature, was used to calculate the ventricular-vascular impedance match (36). Here we focused on the impedance match between the ventricle and vasculature using a formulation previously introduce by us (35), with the general formula for the left impedance matching index given by:

(1) $$\mathrm{Left}\;\mathrm{impedance}\;\mathrm{matching}\;\mathrm{index}=\frac{\mathrm{cardiac}\;\mathrm{time}\times \mathrm{average}\;\mathrm{aortic}\;\mathrm{blood}\;\mathrm{velocity}}{\mathrm{aortic}\;\mathrm{diameter}}$$

Originally, the “cardiac time” variable was the end-systolic duration, but subsequent work (not shown) has demonstrated that the cardiac cycle duration is more appropriate. Using parallel logic we define the right impedance index as;

(2) $$\mathrm{Right}\;\mathrm{impedance}\;\mathrm{matching}\;\mathrm{index}=\frac{\mathrm{cardiac}\;\mathrm{time}\times \mathrm{average}\;\mathrm{main}\;\mathrm{pulmonary}\;\mathrm{artery}\;\mathrm{blood}\;\mathrm{velocity}}{\mathrm{main}\;\mathrm{pulmonary}\;\mathrm{artery}\;\mathrm{diameter}}$$

### 2.3. Linear model generation and optimization

#### 2.3.1. PAPs and SBP models

Candidate variables for the PAPs and SBP linear models were selected from CMR and baseline demographic data. PAPs data were only available for the Complexa PAH cohort, while for the SBP model, data were available from the combined patient cohort, thus more data were available to fit SBP than PAPs. Each candidate variable was correlated separately with the PAPs data and with the SBP data. Additionally, a sine transform of the candidate variable was correlated with PAPs and SBP;

(3) Sinetransformofvariable=Sine(Frequency×Variable+Phase)

Where the Frequency and Phase parameters were optimized by a generalized reduced gradient nonlinear approach implemented in MS Excel to maximize the Pearson correlation r value (37). Variables with a correlation r value greater than 0.2 were entered into the candidate multivariable models. The two multivariable linear models were separately optimized by the systemic search algorithm to maximize each model’s *r*^2^ value by systematically changing each variable’s sine transform Frequency and Phase parameters. In the final linear models, variable entry was allowed at the *p* < 0.05 level. To ensure that highly correlated variables did not inflate the model’s correlation *r*^2^, the variance inflation factor (VIF) was calculated, and only variables with a VIF < 5 were retained. The linear model variables together with the sine transform variables are shown in Table 3 for both PAPs and SBP linear models.

**Table 3:** PAPs and SBP linear and sine transform components.

PAPs model	Sine frequency	Sine phase	Model coefficient (95% confidence interval)	*p* Value	VIF
**Linear variables**					
Intercept			74.76 (63.05–86.47)	<0.001	0
Age			−0.68 (−0.89 to 0.47)	<0.001	1.37
RV mass			0.39 (0.31–0.46)	<0.001	1.43
**Sinusoidal transformed variables**					
LVEF	0.33	0.36	−8.75 (−12.46 to 5.03)	<0.001	1.53
RVEF	1.25	2.79	−7.35 (−11.24 to 3.45)	<0.001	1.35
LV end systolic volume	0.90	−5.13	−6.75 (−10.3 to 3.19)	<0.001	1.27
RV end systolic volume	1.16	0.62	−11.74 (−15.28 to 8.21)	<0.001	1.16
LV impedance slow	0.98	1.91	13.65 (9.98–17.33)	<0.001	1.16
LV impedance rapid	2.75	−1.80	9.71 (6.1–13.33)	<0.001	1.33
RV impedance slow	1.91	−1.54	4.94 (0.98–8.89)	<0.05	1.21
**SBP model**					
**Linear variables**					
Intercept			103.62 (92.13–115.11)	<0.001	0
age X			0.45 (0.29–0.6)	<0.001	1.09
LV mass X			0.17 (0.09–0.25)	<0.001	1.29
RV mass X			−0.26 (−0.32 to 0.19)	<0.001	1.25
**Sinusoidal transformed variables**					
LVEF	0.70	−2.87	−8.87 (−12.42 to 5.31)	<0.001	1.2
RVEF	1.35	−5.99	5.57 (1.98–9.17)	<0.001	1.21
LV end systolic volume	0.98	−4.98	−9.55 (−13 to 6.09)	<0.001	1.1
RV end systolic volume	1.19	1.02	−7.63 (−11.23 to 4.03)	<0.001	1.17
LV impedance slow	1.14	0.34	7.78 (4.13–11.42)	<0.001	1.27
LV impedance rapid	2.57	−1.08	−8.88 (−12.57 to 5.18)	<0.001	1.15
RV impedance slow	2.09	−4.08	−19.31 (−25.25 to 13.38)	<0.001	3.73
RV impedance rapid	1.94	−0.51	−19.13 (−25.37 to 12.89)	<0.001	3.8

#### 2.3.2. 6MWD model

To generate the multivariable model of the 6MWD for the Complexa PAH patients, candidate variables were selected based on a suitably high correlation *r* with the 6MWD either for the linear variables or the sinusoidally-transformed variables. Similar to the blood pressure models, the parameters of the sine transforms were selected by optimizing the correlation *r*^2^ value by the automatic search algorithm.

#### 2.3.3. Linear pressure relationship

To illustrate the nature of the relationship between SBP and PAPs we conducted a simulation using the linear models, whereby an artificially constructed set of parameters of the SBP model was generated to achieve a target SBP. Here two separate SBP targets were simulated: 120 and 140 mm Hg. Since multiple variables contribute to each pressure condition, multiple combinations of parameter values can achieve the same target SBP. In our case 20 combinations of variables were generated to achieve each target pressure. These variables were then entered into the PAPs linear model to generate the corresponding simulated PAPs data and the results were plotted using a box plot.

### 2.4. Mean and pulsatile blood pressure component representation

To aid in developing an intuitive understanding of the PAPs and SBP model components we combined pressure components to relate to the concept of hydraulic power. Conventionally, the steady hydraulic power is calculated as mean PAP × cardiac output while the remaining power component is associated with pulsatile blood flow (38). Hydraulic power is rarely measured since it requires simultaneous measures, time-resolved thought the cardiac cycle, of high-fidelity pressure and flow data (39). Here we assigned the sinusoidal components to the pulsatile hydraulic power component since the sinusoidal transform is integral to the time-varying aspect of ventricular-vascular interaction. The linear components were assigned to the mean hydraulic power since they contain only time-invariant components. Thus, four separate components of hydraulic pressure were generated:

(4) Hydraulicpulmonarypulsatile(HPp)=sumofpulmonarysinusoidalcomponents

(5) Hydraulicpulmonarymean(HPm)=sumofpulmonarylinearcomponents

(6) Hydraulicsystemicpulsatile(HSp)=sumofsystemicsinusoidalcomponents

(7) Hydraulicsystemicmean(HSm)=sumofsystemiclinearcomponents

### 2.5. Statistical analysis

Count statistics were represented as number and percentage, continuous data were analyzed as mean and standard deviation if normally or nearly normally distributed, and as median and interquartile range if non-normally distributed. Comparison between grouped variables was performed with *t*-testing or the non-normal equivalent, as appropriate. Analysis of grouped data were presented as box plots, with interquartile ranges and outliers identified by the “o” symbol. Linear regression modeling was performed using a stepwise forward inclusion approach. To estimate the variability of each linear regression model a bootstrapping approach was applied with 1,000 randomly resampled data sets. The mean and standard deviation of the bootstrapped model correlation *r*^2^ was used to calculate the mean and 95% confidence intervals. For linear regression models that incorporated measured data the fitted regression line was generated by Deming regression that takes into account variation in dependent and independent variables (40). The linear model results were subjected to Bland-Altman analysis to yield the model bias and 95% confidence interval. The relationship between pulmonary and systemic hydraulic pressure components were plotted against increasing values of PAPs with a five-point moving average applied to allow data trends to be more easily visualized. Logistic regression modeling was used to distinguish PH from PAH patients. Discrimination capability of the model was measured by the area under curve (AUC) from a receiver operating characteristic (ROC) curve analysis. For an estimate of sensitivity and specificity a cut-off value was set to approximately equalize sensitivity and specificity. Statistical significance was regarded at *p* < 0.05. Statistical analyses were performed using SPSS 18.0 (SPSS Inc., Chicago, Illinois).

## 3. Results

For the 52 Complexa patients, the demographic and test results at baseline are shown in Table 1. Complete CMR and RHC data were available in 41 (79%) patients. The mean time between the RHC and the CMR examination was 9.5 days with a standard deviation of 11 days. For the 49 patients with a suspicion of pulmonary hypertension (PAH, *n* = 34, 70% or PH, *n* = 15, 30%) who were referred to receive a CMR examination, estimates of PH pressure were available in 17 (35%) with 15 (31%) having a suspicion of PAH. The demographic and test results are shown in Table 2 separately for the PAH and PH groups.

### 3.1. Pressure modelling

**Figure 1:**
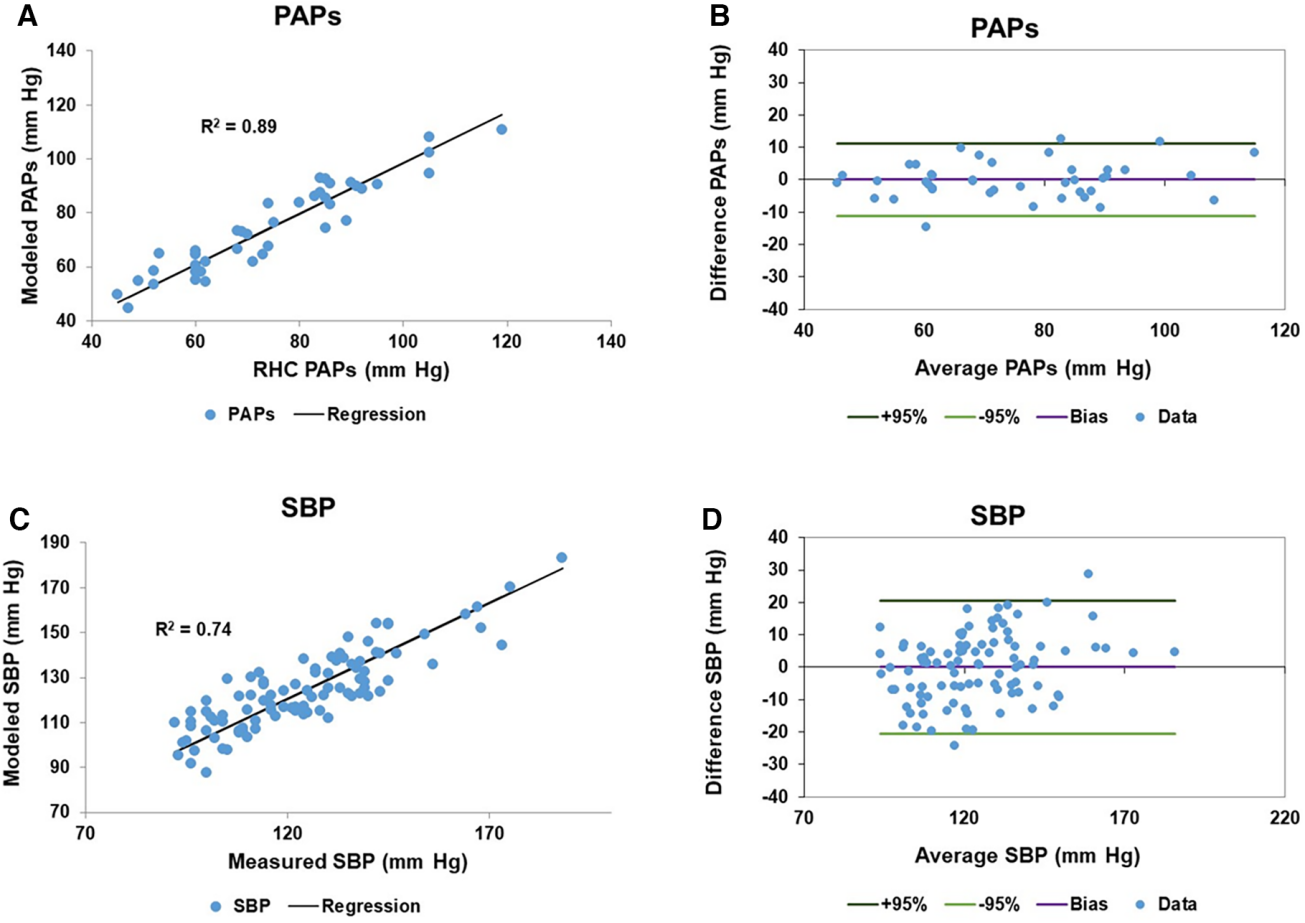
Model results of pulmonary artery systolic pressure (PAPs) and systemic systolic blood pressure (SBP): (**A**) the scatter plot of the right heart catheter (RHC) measured and fitted PAPs data has a regression *r*^2^ of 0.89. (**B**) The corresponding Bland-Altman plot of the fitted and measured PAPs data has a bias is zero and the 95% confidence limits of agreement are ±11.2 mm Hg. (**C**,**D**) Are the corresponding scatter and Bland-Altman plots for the measured and fitted SBP data, respectively. The measured and fitted SBP data has a regression *r*^2^ of 0.74, while the Bland-Altman bias is zero and the 95% confidence limits of agreement are ±20.5 mm Hg.

Only patients with RHC measures of PAPs (Complexa cohort) contributed to the linear model of PAPs. To generate the linear model of SBP, patients from both cohorts were entered in to the linear model. Each linear model included cardiac component variables from both right and left ventricles. In the case of left and right impedance match values we identified two sine transforms, one termed slow (lower frequency) and the other rapid (higher frequency) which were sufficiently independent for inclusion in the model since the VIF was <5. Table 3 itemizes the cardiac variables included in each linear model, including the sine transform Frequency and Phase parameters, the linear coefficient and 95% confidence interval, *p* value and VIF are noted. Age was the only non-CMR-determined variable. Importantly, the models did not contain any indication of which patients had PAH vs. PH. The fitted and modeled PAPs and SBP data were evaluated using linear regression and Bland-Altman analysis, Figure 1. The results of bootstrapping the models are: SBP model *r*^2^ = 0.77 (95% CI: 0.64–0.91) and PAPs model *r*^2^ = 0.92 (95% CI: 0.85–0.99).

### 3.2. Pressure relationships

**Figure 2:**
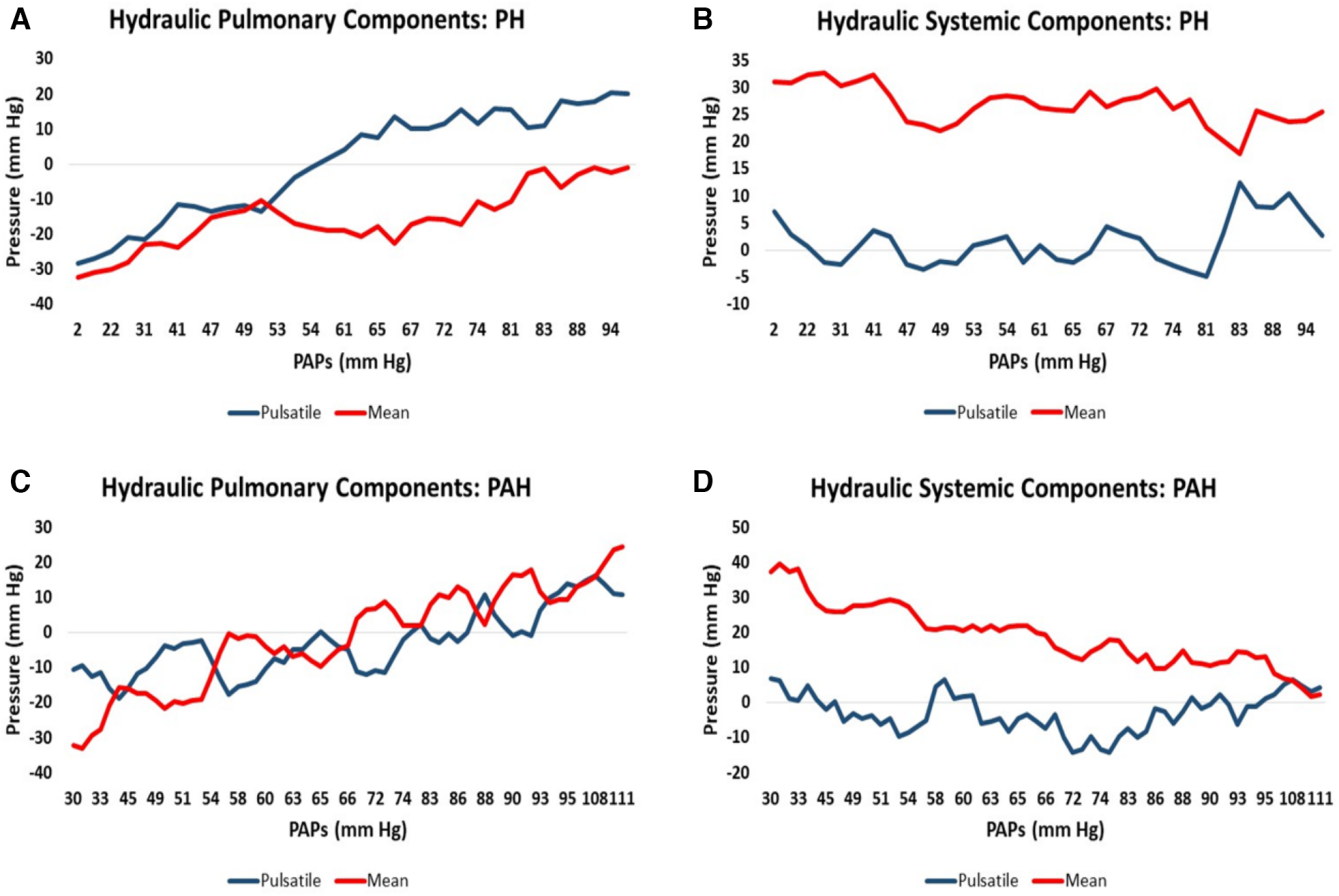
Moving average of hydraulic pressure components for pulmonary hypertension (PH) and pulmonary arterial hypertension (PAH) patients plotted against increasing pulmonary artery systolic pressure (PAPs): (**A**) the hydraulic pulmonary pulsatile (HPp) and hydraulic pulmonary mean (HPm) pressure for PH patients. (**B**) The corresponding hydraulic systemic pulsatile (HSp) and hydraulic systemic mean (HSm) pressure plots for PH patients. (**C**,**D**) Are the corresponding pulmonary and systemic data plots for PAH patients, respectively.

Since the suspected PH/PAH patient group did not have RHC-measured PAPs contemporaneously with CMR we uniformly employed the modeled PAPs data to plot against each component of hydraulic pressure. Patients from this group were separated into PH (*n* = 34, 70%) and PAH (*n* = 15, 30%) to allow the moving average of the two pulmonary and two systemic hydraulic pressure components to be plotted separately for PH and PAH patients, Figure 2. The 34 PH patients were drawn exclusively from the suspected PH/PAH cohort, while the PAH patients comprised Complexa (*n* = 52) and an additional 15 patients (total *n* = 67) from the suspected PH/PAH cohort. Pressure data from linear models are plotted without regard to static offsets.

**Figure 3:**
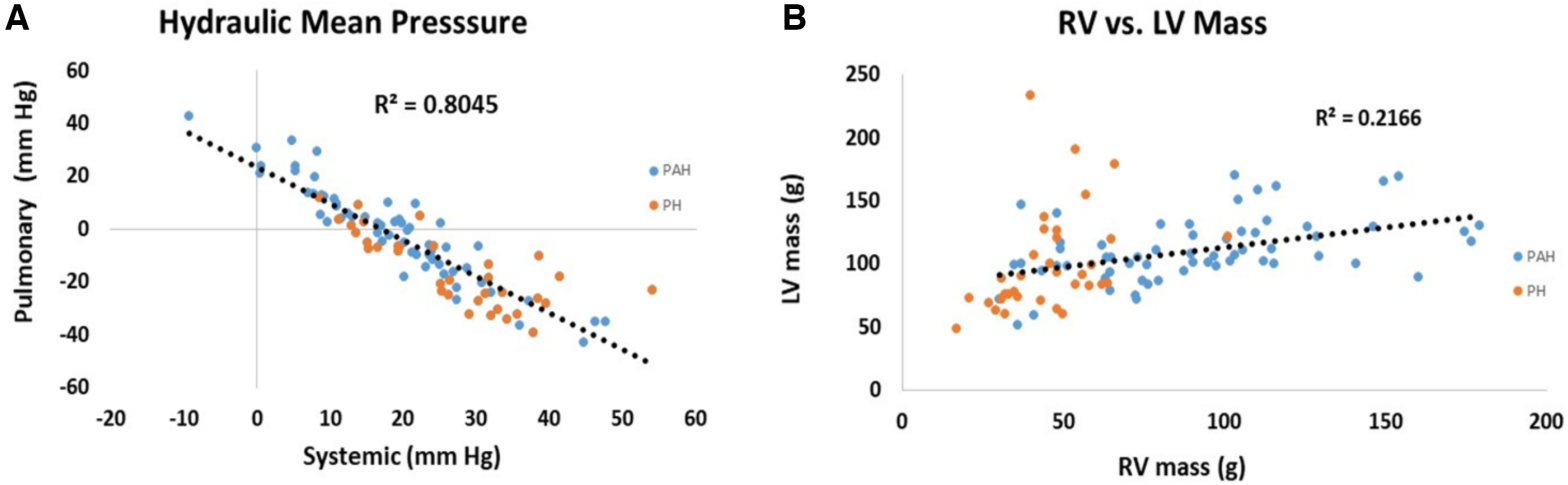
Scatter plots of (**A**) hydraulic pulmonary mean pressure (HPm) vs. hydraulic systemic mean pressure (HSm) and (**B**) scatter plots of right ventricular (RV) mass vs. left ventricular (LV) mass. Points are plotted separately for pulmonary arterial hypertension (PAH) patients (blue) and pulmonary hypertension (PH) patients (orange). Note, that mean pressure components are strongly inversely related (*r*^2 ^= 0.80) while ventricular masses are weakly positively correlated (*r*^2^ = 0.22).

**Figure 4:**
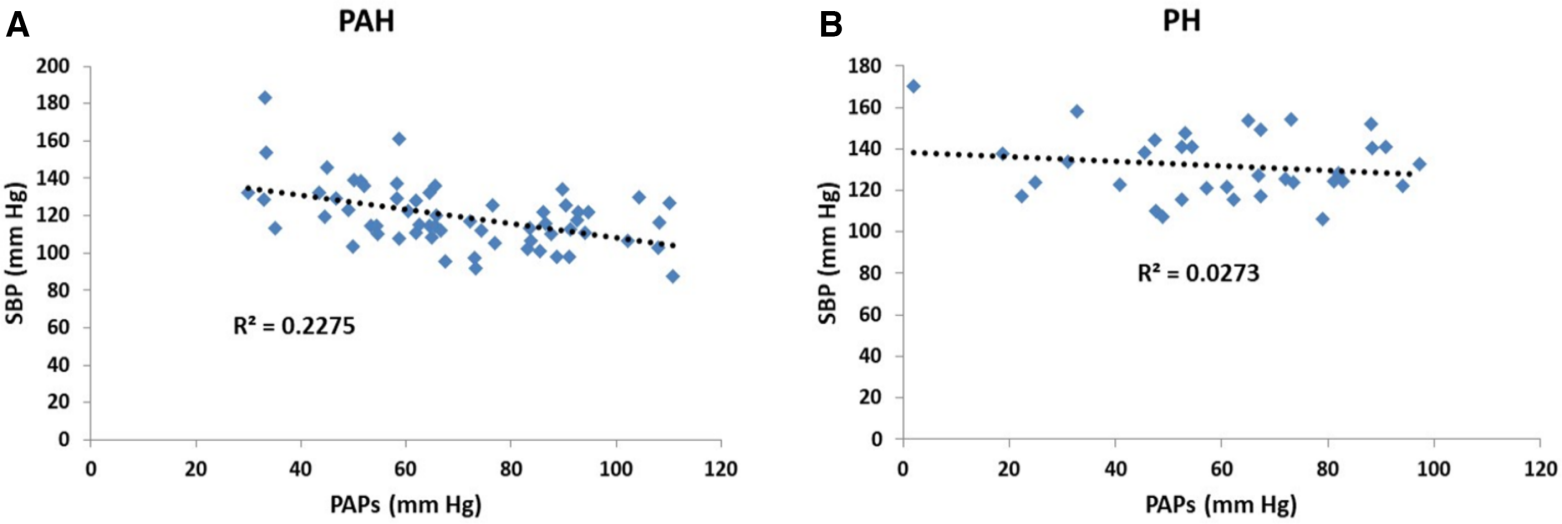
Scatter plots of (**A**) systolic blood pressure (SBP) vs. pulmonary arterial systolic pressure (PAPs) for patients with pulmonary arterial hypertension (PAH) and (**B**) corresponding scatter plots for patients with pulmonary hypertension (PH). In the PAH patients the pressures are weakly negatively related (*r*^2^ = 0.23) while for PH patients there is no significant trend (*r*^2^ = 0.03).

Scatter plots of the relationship between pulmonary and systemic hydraulic mean pressure components for PAH and PH patients are shown in Figure 3A. Note that for both PAH and PH patients there is a strong negative relationship between hydraulic pulmonary mean pressure and hydraulic systemic mean pressure, with the PAH data spread over a wider range compared to PH. In Figure 3B the scatter plot of right and left ventricular masses are plotted for PAH and PH patients, displaying a weak positive relationship. Scatter plots of the modeled PAPs and SBP for PAH and PH patients are shown in Figures 4A,B, respectively. Despite the strong negative correlation of hydraulic pulmonary mean and hydraulic systemic mean pressures there is only a weakly negative correlation between PAPs and SBP for PAH and PH patients.

### 3.3. Pressure components differences between PAH vs. PH Patients

**Figure 5:**
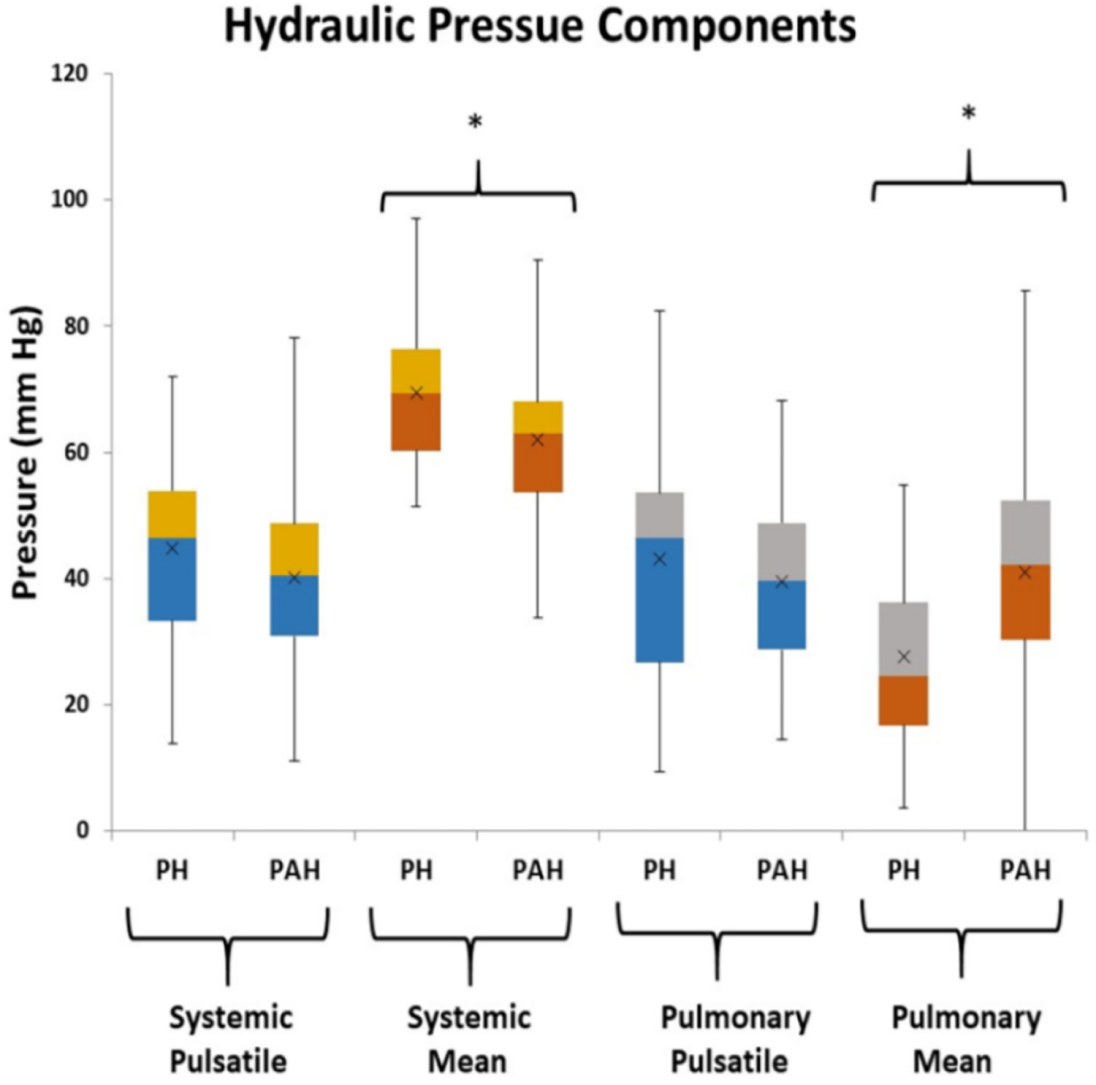
Box plots of the hydraulic pressure components for pulmonary hypertension (PH) and pulmonary arterial hypertension (PAH) patients (the asterisk indicates *p* < 0.05 between PH and PAH). The hydraulic systemic pulsatile pressure (HSp) and hydraulic pulmonary pulsatile pressure (HPp) are not significantly different between groups, while the hydraulic systemic mean pressure (HSm), and hydraulic pulmonary mean pressure (HPm) components are statistically different between groups.

**Figure 6:**
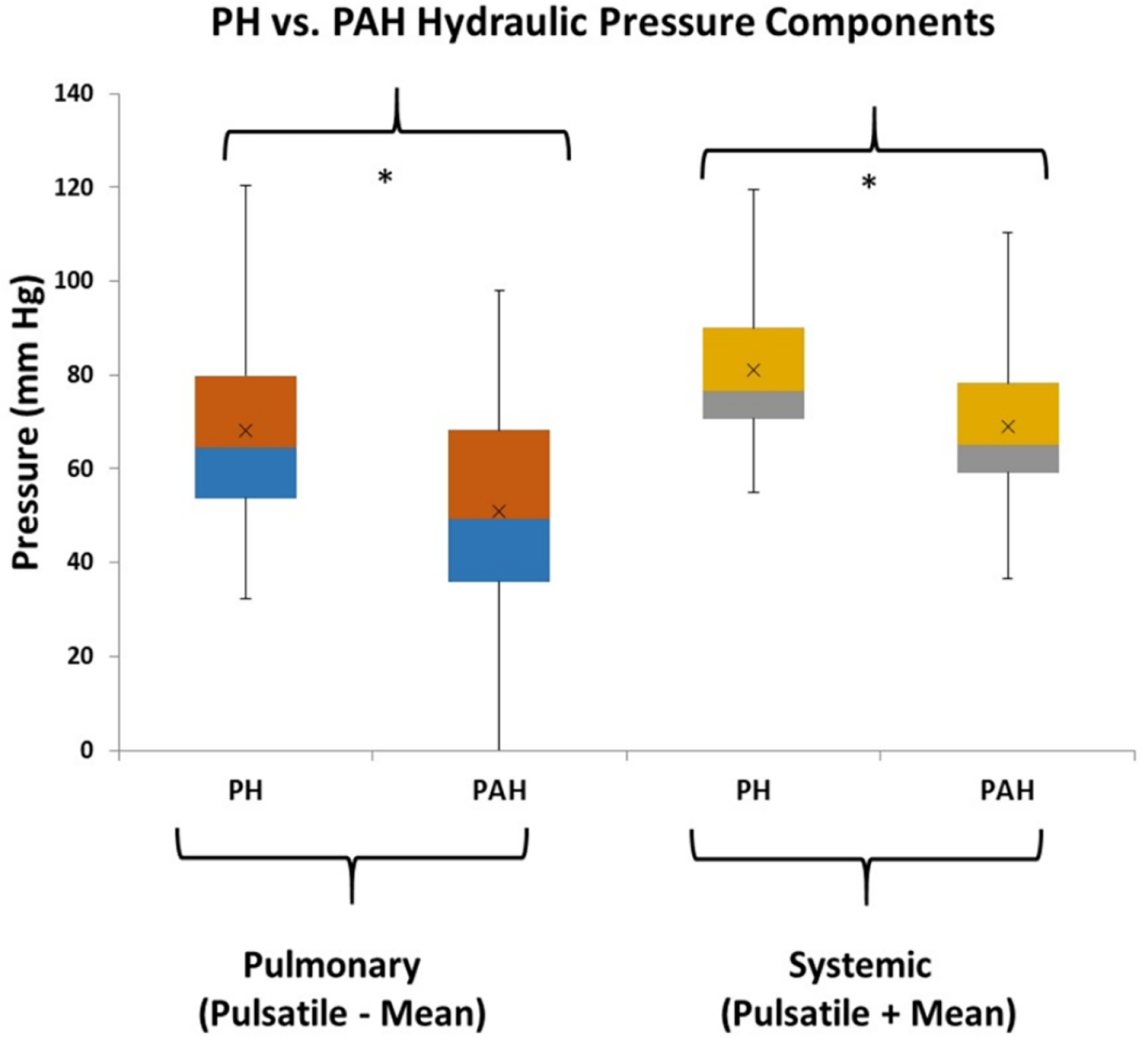
Box plots of combinations of hydraulic components that are different between pulmonary hypertension (PH) and pulmonary arterial hypertension (PAH) patients (the asterisk indicates *p* < 0.05). The pulmonary combination that is different between patient groups is the subtraction of pulmonary hydraulic mean (HPm) pressure from the pulmonary hydraulic pulsatile (HPp) pressure. The systemic combination that is different between patient groups is the summation of systemic hydraulic mean (HSm) pressure and the systemic hydraulic pulsatile (HSp) pressure.

**Figure 7:**
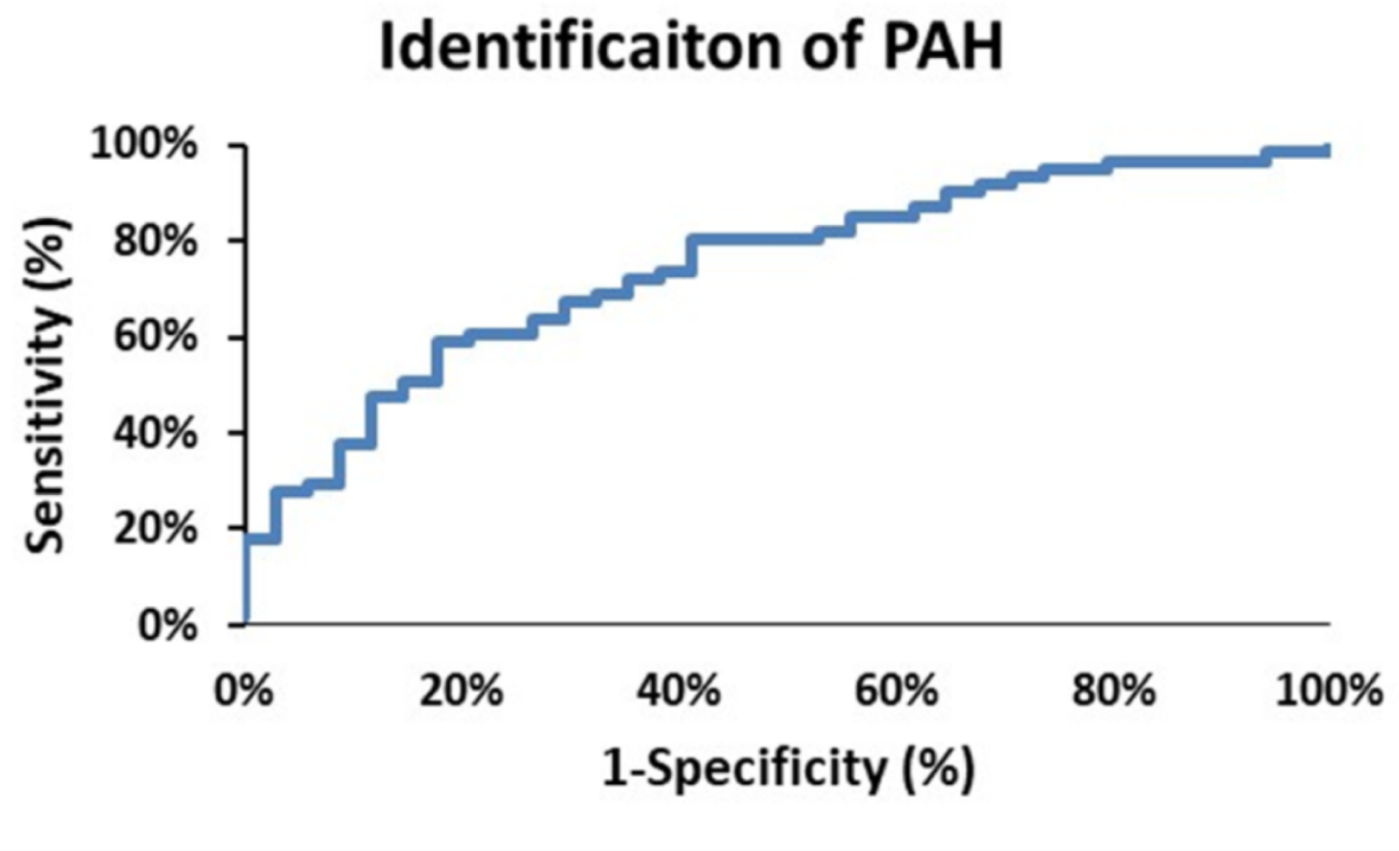
The receiver operator characteristic curve plot for identification of pulmonary arterial hypertension (PAH) patients. The area under the curve is 0.75, and selecting a threshold of 0.66 yields a sensitivity of 68% and a specificity of 68%.

From Figure 2 there are clearly discernible differences in the general pattern of pressure components between PAH and PH patients. These differences are visible over a wide range of PAPs and also in the pressure component’s mean and distribution, Figure 5. We explored what combination of hydraulic pressure components (without averaging) differentiated PAH vs. PH patients. In Figure 6 we show the box plot of two composite pressure components that are statistically different between PAH and PH patients: (A) the difference between hydraulic pulmonary pulsatile and hydraulic mean pressures and (B) the summation of the hydraulic systemic pulsatile and hydraulic mean pressures. These two composite variables were both significant *p* < 0.05) in a binary logistic regression model to distinguish between PAH and PH patients. The corresponding ROC plot had an area under the curve of 0.75, Figure 7. Setting the threshold to 0.66 resulted in a sensitivity of 67% with a specificity of 68%.

**Table 4:** 6 min walk distance linear and sine transform components.

6MWD components	Sine frequency	Sine phase	Model coefficient (95% confidence interval)	*p* Value	VIF
**Linear variable**					
Intercept			−36,110.66 (−65,395.92 to 6,825.4)	<0.05	0
Weight (kg)			−1.17 (−1.95 to 0.38)	<0.001	1.15
Age (years)			−2.41 (−4.24 to 0.57)	<0.05	1.08
**Sinusoidal transformed variables**					
Height (cm)	0.005	0.74	40,166.58 (11,031.79–69,301.38)	<0.05	1.05
Hydraulic pulmonary pulsatile (mm Hg)	0.005	1.51	−3,405.97 (−6,629.03 to 182.91)	<0.05	1.08
RV impedance match	1.16	1.83	−40.63 (−67.39 to 13.87)	<0.001	1.06

### 3.4. Right ventricular pulsatile components

**Figure 8:**
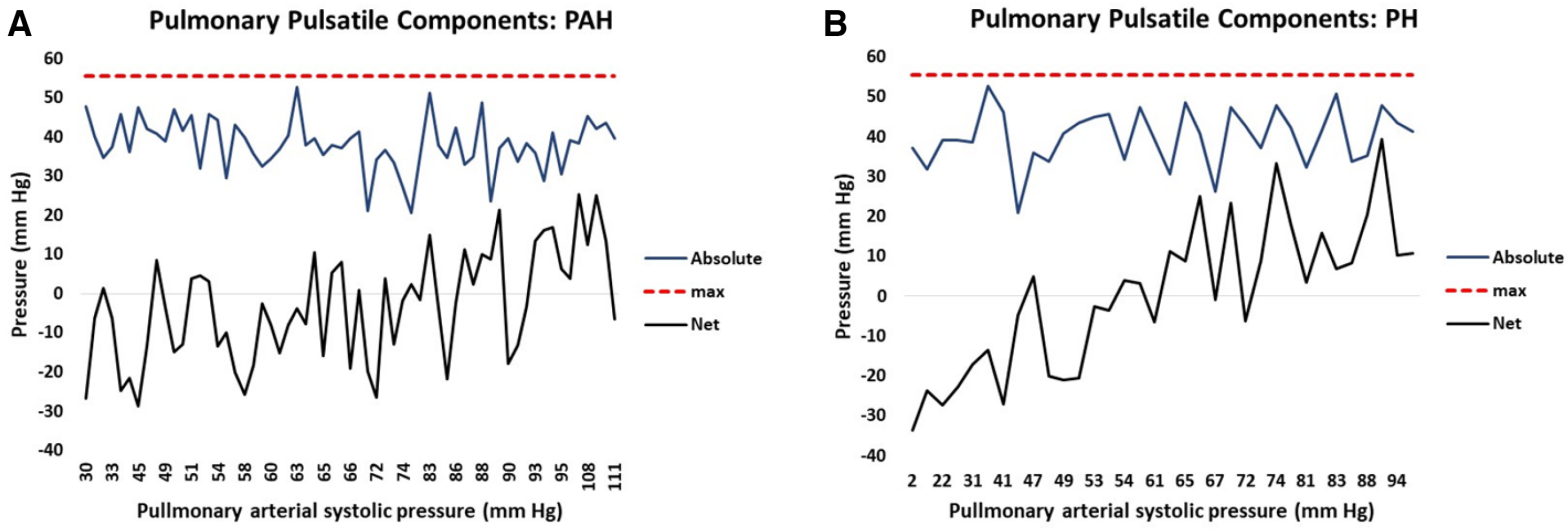
The attained net (black) and potential (blue) hydraulic pulmonary pulsatile pressure component plotted against increasing pulmonary arterial systolic pressure (PAPs) for (**A**) pulmonary arterial hypertension (PAH) patients and (**B**) pulmonary hypertension (PH) patients. The solid black line represents the net summation of each contributing variable of hydraulic pulmonary pulsatile pressure taking into account the phase (i.e. positive or negative contribution) of each variable. The solid blue line represents the net summation of each contributing variable of hydraulic pulmonary pulsatile pressure without taking into account the phase (i.e. each contribution is positive) of each variable. The punctuated red line represents the model-determined maximum value of hydraulic pulsatile pressure (56 mm Hg).

The right ventricular hydraulic pulsatile component is formed from the summation of seven sine-transformed variables. The sine transform varies systematically from positive one to negative one and is further multiplied by a scaling factor for each variable. Thus, summation of the absolute magnitude of each sine-transformed variable identifies the maximum possible hydraulic pulmonary pulsatile value, i.e., corresponding to the condition when all sine contributions are maximally positive. In this case the maximum possible pulsatile pulmonary value is 56 mm Hg which represents the upper limit of the hydraulic pulmonary pulsatile component. Thus, the linear model of PAPs naturally produces the concept of RV contractile reserve, which we define as the difference between the current hydraulic pulmonary pulsatile value and the upper limit of 56 mm Hg. For PAH patients, the plots in Figure 8A show how the seven contributions add to produce the realized net and theoretical absolute maximum hydraulic pulmonary pulsatile component. Due to the mixture of positive and negative contributions (depending on the phase of each variable’s sine wave) the realized net hydraulic pulmonary pulsatile pressure value (black line) ranges from negative through positive with increasing PAPs. However, by discarding the phase of each contribution (i.e., considering the absolute magnitude of each variable’s contribution) the sum of each component of hydraulic pulmonary pulsatile component produces the theoretical absolute value (blue line). Note that the absolute summation of each of the seven variable’s contributions do not systematically vary over the range of PAPs. The corresponding net and theoretically absolute maximal amplitudes of hydraulic pulmonary pulsatile pressure are plotted in Figure 8B for PH patients, respectively.

**Figure 9:**
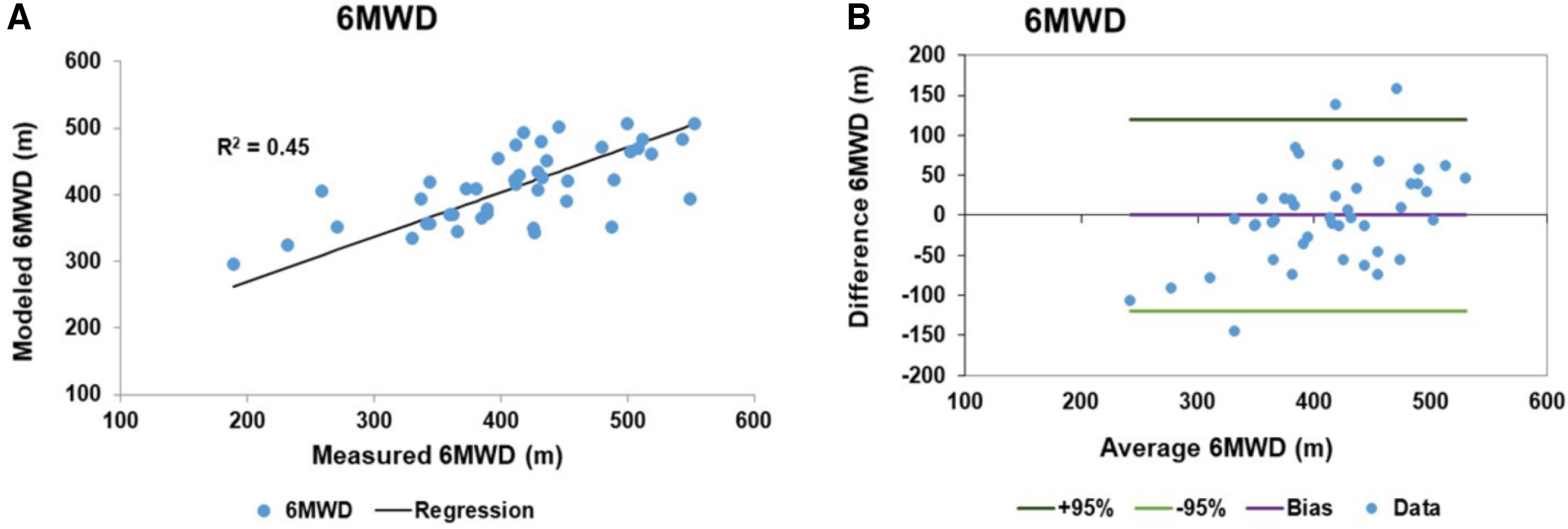
Linear model results of 6 min walk distance (6MWD): (**A**) the scatter plot of the 6MWD measured and fitted data with a regression *r*^2^ of 0.45. (**B**) The corresponding Bland-Altman plot of the fitted and measured 6MWD data. The bias is zero and the 95% confidence limits of agreement are ±119 m.

The RV functional reserve is known to be a factor in determining the 6MWD along with biomechanical variables (41). We generated a linear model to predict the 6MWD in PAH patients which included the biomechanical variables of height, weight and age and the pulmonary pressure variables of hydraulic pulmonary pressure and the RV impedance match. Height, hydraulic pulmonary pressure and RV impedance match were related via sinusoidal transforms while weight and age were linearly related. Sine transform variables and model coefficients are given in Table 4. The linear model accounted for 45% of the variation in the 6MWD. The scatter plot of modeled and measured 6MWD is shown in Figure 9A with the corresponding Bland-Altman plot in Figure 9B. The bias term is zero and the 95% confidence limits are ±119 m.

### 3.5. Pressure simulation

**Figure 10:**
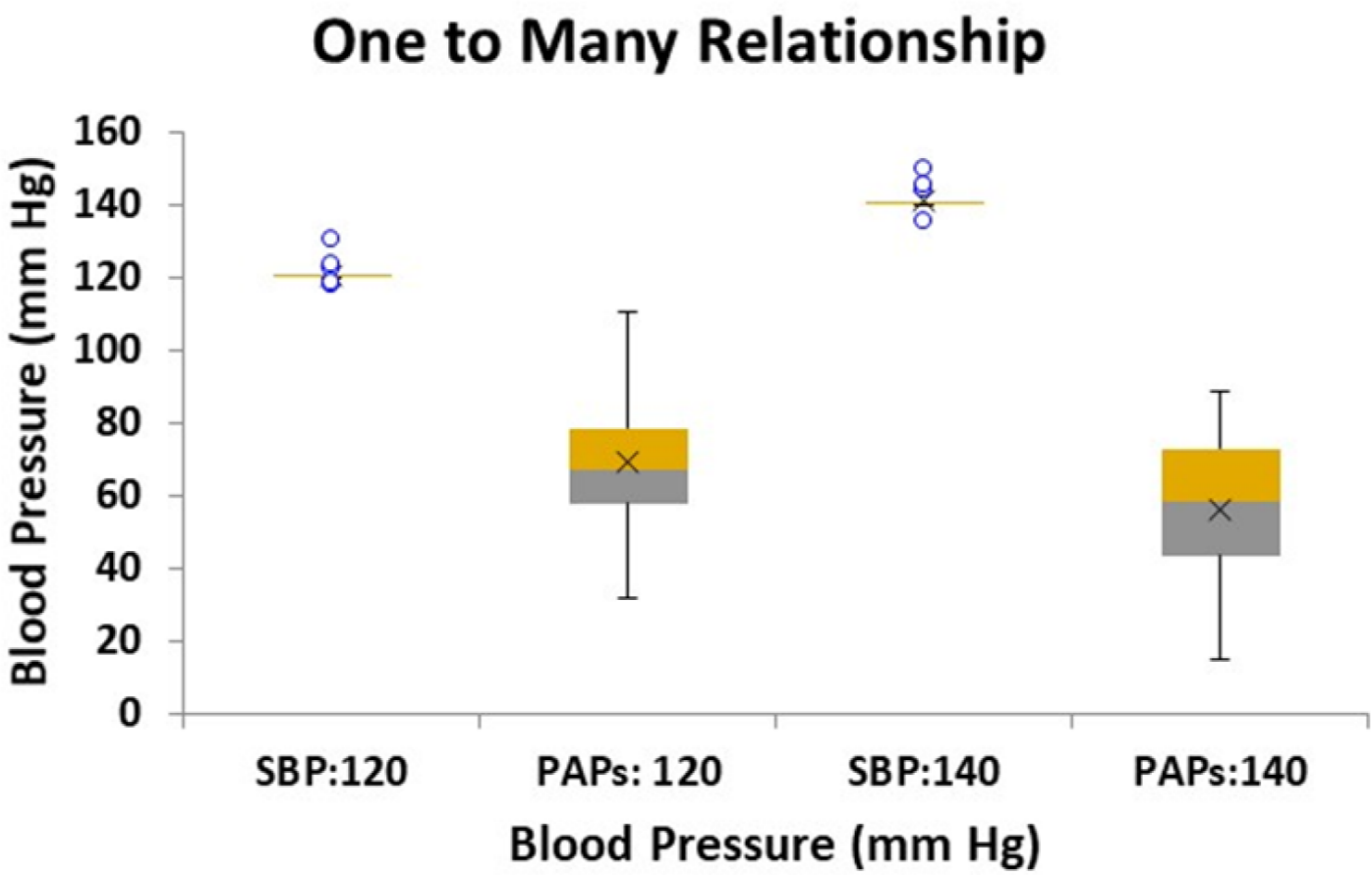
Box plot of simulated linear model results of conditions relating to two target systemic systolic blood pressure (SBP) levels: 120 and 140 mm Hg with the corresponding modeled systolic pulmonary artery pressure (PAPs) range of pressures. Twenty sets of conditions were modeled for each target SBP. The PAPs corresponding to SBP target of 120 mm Hg is centered on 69.1 mm Hg with a standard deviation of 4.0 mm Hg, while the PAPs range corresponding to SBP target 140 mm Hg is centered on 55.8 mm Hg with a standard deviation of 4.7 mm Hg, (*p* < 0.05).

The results of the pressure simulations for the two targeted SBP levels of 120 and 140 mm Hg are shown in the box plots of Figure 10. Here we see that for each target SBP (employing 20 sets of artificially generated parameters) the same set of parameters resulted in a wide range of PAPs values. Note that the lower SBP target of 120 corresponds to the higher PAPs data centered on 69 (SD 18) while the higher SBP target of 140 corresponds to the lower PAPs data centered on 56 (SD 21, *p* < 0.05). The lower and higher set of PAPs values were statistically different (*p* < 0.05) and clearly show that each target value of SBP produces a range of values of PAPs, i.e., a one to many relationship.

## 4. Discussion

We introduced two linear models that quantified the relationships that exists between the pulmonary and systemic pressure systems and which further show the nature of the interrelationships of the right and left ventricles. In particular, these interrelationships were demonstrated in a cohort of well documented PAH patients which met all WHO criteria, including the stipulation of hypertension not being secondary to left-sided disease. We interpreted the pressure component derived from linear variables as an index of mean hydraulic power and the pressure component derived from the sine-transformed variables as an index of pulsatile hydraulic power. For the combined patient cohort, which included PAH and PH patients, the linear models showed how the separate hydraulic pressure components varied with increasing PAPs and demonstrated that they exhibited distinct patterns and values that distinguished between PAH and PH patients with good sensitivity and specificity. The concept of right ventricular reserve naturally arose from the model with the reserve index significantly correlating with the 6MWD.

There are several features of the models that are consequences of the construct adopted, namely that the heart conditions represent the state variables of the system. The concept of state variables is more natural to engineering than physiology, whereby the behavior of a dynamic system comprised of multiple parts (e.g., the breaking system of an automobile) can be characterized by a small number of state variables (e.g., the vehicle speed and road-tire coefficient of friction) (42). In the case of the heart, the state variables are weights (ventricular mass), volumes (ESV) and efficiency ratios including EF and the coupling efficiency at the interface to the vasculature (impedance match). The state space representation allows determination of the response of the system to a time-dependent action (e.g., in the case of the breaking system, the rate of application of the break). In the case of the heart the non-linear variables represent the state of the heart at end-systole and the sinusoidal transforms are related to the time course over the cardiac cycle of each variable. The sinusoidal time course was postulated from the observation of Suga and Sugawa concerning the time course of pressure generation in the ventricle. An important aspect of the state-space representation is that external conditions affect how the system behaves (e.g., in the case of the breaking system, icy road vs. dry road conditions). In the case of PAH vs. PH the external conditions are pre-capillary vs. post-capillary elevated resistance/impedance, respectively, and these conditions dramatically affect the response of the heart. The right and left ventricular masses are static over the time course of the cardiac cycle and were thus assigned as contributors to mean pressure. The only non-cardiac variable was age, Table 3. While it has been established that increasing age is associated with higher levels of PAPs in the general population (43) surprisingly the model coefficient of age was negative. However, while the positive association with age can be observed in the general population, here we considered a population with confirmed PAH or a high suspicion of PH/PAH and applied a model that adjusts for a number of cardiac variables which may in turn be age dependent. There are many potential applications of the model: (1) since all model variables are non-invasively obtained, clinical monitoring of PAH patients can be achieved without conducting a RHC, (2) since multiple variables contribute to each model the detailed effects of a therapy on each component can be assessed in an ongoing manner, (3) all model measurements are performed in the heart, and while the heart might not be the target of a therapy the effects of external conditions ultimately manifest in the heart, (4) the models provide estimates of pulmonary and systemic conditions, (5) since the PAH population is small, and does not easily lend itself to large randomized clinical trials (44), there is the possibility that trials might be designed to observe the effects of each therapy in PH patients to suggest how they might transfer to PAH patients and *vice versa* (45).

### 4.1. Model accuracy

From Figures 1A,B we see that the model accounted for 89% of the variation of PAPs. The Bland-Altman analysis showed that the 95% confidence limits were ±11 mm Hg, which is on the order of the reported spontaneous variation of PAPs of 20%–25% (46). We did not have reliable contemporaneous measures of PAPs for patients in the suspected PH/PAH cohort. However, the available estimated PAPs values were compared to the modeled values using a paired t-test which failed to reject the hypothesis that the measurements were different (*p* = 0.40). Others have shown that the use of CMR in studying PAH patients has excellent repeatability and registered a larger therapy effect size than either the 6MWD or N-terminal pro b-type natriuretic peptide (47).

The linear model of SBP accounted for 74% of the variation despite having considerably more patients contributing to the model. However, it has been noted that the typical clinical measurement of SBP is notoriously inaccurate (48, 49). Consider a hypothetical example where the standard deviation of measuring the SBP is on the order of 5 mm Hg. Under this scenario a person with a nominal SBP reading of 125 mm Hg (i.e., pre hypertensive) could well be hypertensive (upper 95% confidence level 135 mm Hg) or normotensive (lower 95% confidence level 115 mm Hg) (50). However, in routine clinical practice the standard deviation of SBP ranges from 14 to 26 mm Hg depending on measurement method and personnel (34, 51). The lower range of the clinical standard deviation is comparable to the standard deviation obtained here for the model SBP Bland-Altman analysis of 10.5 mm Hg. Thus, we believe that the lower level of agreement between modeled and measured SBP in part reflects the difficulty of obtaining a reliable measure of SBP.

### 4.2. Heart failure in PAH vs. PH

It is widely accepted that the mode of death in PAH and PH patients is predominantly due to RH-failure (31, 52). However, heart failure remains a clinical syndrome with little indication of the details of how failure occurs (53). Examination of the relative pressure components may indicate differences in the mode of heart failure that are expected in PAH vs. PH. Figure 2C shows that in PAH patients both hydraulic pulmonary mean and pulsatile components increase in a broadly parallel manner with increasing PAPs. We speculate that this is due to the normally widely-distributed compliance of the pulmonary arterial tree becoming progressively concentrated in the pulmonary artery, such that the RV ejection pattern progressively resembles that of the LV, where 80% of the systemic compliance is localized in the aorta (54). Thus, as PAH progresses, the hydraulic pulmonary mean pressure increases, but since this is largely contributed to by RV mass (Table 3) at some point the heart may be physically limited in its ability to increase the hydraulic mean pressure, leading to RH failure. Figure 2D shows the that hydraulic systemic mean pressure steadily declines as disease progresses, consistent with the clinical observation that widespread end-organ damage is sustained due to low perfusion pressure (55). A careful study of the mode of death of PAH patients found that only 50% definitively suffered from RH-failure, while the remaining 50% died in the ICU (56), conceivably due to left-sided complications. Thus, interpretation of Figures 2C,D are consistent with observations concerning the changes in physiology, types of morbidity and modes of death observed in PAH patients.

In PH patients, as disease progresses the normally highly compliant pulmonary artery progressively loses compliance, which in turn requires the RV to increase pulse pressure (57). The pulmonary conditions for PH patients are shown in Figure 2A where there is an early elevation of both hydraulic pulmonary pulsatile and mean pressure components, but when PAPs exceeds about 50 mm Hg, the pulsatile component preferentially increases. This in turn causes the reflected pulse pressure wave to rapidly arrive back at the right ventricle prior to closing of the pulmonic valve, further opposing ventricular ejection during late systole (58). However, the model indicates that the increase in hydraulic pulmonary pulsatile pressure cannot proceed beyond an upper limit of 56 mm Hg. With reference to Figure 8A it can be appreciated that in advanced stages of PH the RV pulsatile component approaches the maximum value, i.e., approaching the condition of zero RV pulsatile reserve. This indicates that PH patients may be prone to RV failure due to an inability to increase the hydraulic pulmonary pulsatile component, which is indirectly supported by the association of higher mortality with increased pulse pressure (59). It has been noted that in patients with PH secondary to left-sided heart failure with preserved EF that the degree of loss of RV function greatly exceeds that of the LV (19, 60). In Figure 2B it is apparent that the systemic pressure adaptations are not as dramatic as those of the RV (Figure 2A). Thus, the model reveals several differences in pressure components between PAH and PH which may be explanatory of the different modes of heart failure and whether right or left ventricles are likely to fail or result in morbidity.

### 4.3. Interconnectedness of ventricular responses

The high-degree of interconnectedness of the pulmonary and systemic pressure systems that is demonstrated by the linear models may be a controversial aspect, especially in PAH patients where the role of the left ventricle may be generally underappreciated (61). While right ventricular changes gradually accrue, clinically relevant changes to the left ventricle tend to manifest at the end stage (62), leading to the concept put forth of the forgotten left ventricle in right ventricular pressure overload (63). In advanced PAH the left ventricle has been clinically noted to appear “small and underfilled” and hyperdynamic (20, 64, 65). This issue directly relates to the underlying determinants of ventricular responses to PAH (66). Figure 3A shows the close to perfect antisymmetric relationship between pulmonary and systemic mean hydraulic pressure components. If the determinant of ventricular response was due to circulating neurohumoral factors, a symmetric response of each ventricle would be expected. Conversely, if the ventricles were independent and responded to localized workloads they could be positively or negatively related, but the relationship would not be expected to be strong due to their independence. However, if the ventricles were interdependent then the response would be expected to be strongly negative, i.e., antisymmetric, which is what the model indicates. Note, that the strong anti-symmetric relationship between hydraulic pulmonary mean and hydraulic systemic mean pressure is not a feature forced by the linear models. Further, Figure 3B shows that RV mass exhibits a weak positive correlation with LV mass which, given the constrained total volume, indicates a reduction in LV chamber volume consistent with the small and underfilled LV. This reduction in LV afterload is expected to weaken the LV myocytes, an expectation that was confirmed in a recent study conducted in PAH patients undergoing cardiac transplantation where it was found that left ventricular myocytes were thinner and exhibited a reduced force generating capacity compared to those of donor hearts (67). The weaker LV myocytes indicate a reduced ability to sustain a high hydraulic systemic mean pressure, which is only partially compensated for by an increased mass. Thus, the exhibited ventricular interdependence of mean pressure is consistent with clinical and physiologic observations.

One may counter that for PAH patients, while there is evidence of left-sided dysfunction (68), the literature does not indicate that there is a clear inverse relationship between pulmonary and systemic pressure, with some observations even indicating a weak positive correlation (69, 70). Figure 4A indicates that the linear models established a weakly negative relationship between PAPs and SBP. Several aspects of the linear models explain why this is the case. Firstly, Figure 10 shows that the linear models do not predict a one-to-one relationship between SBP and PAPs. In the simulated results of Figure 10 several sets of parameter combinations were applied to generate a SBP close to 120 mm Hg which resulted in generating a wide range of PAPs values centered on 69 mm Hg. Conversely, the simulation for the higher SBP value of 140 mm Hg generated a range of wide PAPs values centered on the lower value of 56 mm Hg. That is, there is a one-to-many relationship between SBP and PAPs while the overall trend is negative. Secondly, while there is a strong asymmetric relationship observed between the hydraulic systemic mean and hydraulic pulmonary mean pressures, we note that these are calculated components of the model and may not correspond to any easily-measured pressure component. Thirdly, the difficulty in obtaining accurate measures of SBP requires large numbers of patients to observe these trends. As has been noted, systemic pressure conditions in PAH are widely underappreciated and underreported in sufficient detail to allow determination of the relationships by performing Meta-analysis. Further, commonly applied therapeutic interventions in PAH patients may reduce left-sided pressure (55) and thus any relationship observed may be interpreted as being a side effect of medication.

### 4.4. Ventricular reserve

Right ventricular output reserve is the ability of the RV to increase output in response to acute exercise or pharmacologic stress. RV reserve can be accessed via multiple indices including cardiac output, pulmonary vascular resistance, pulmonary capacitance, tricuspid annular plane systolic excursion and pulmonary artery pulsatile pressure (71, 72). A common feature of all cardiac reserve assessments is the requirement for a comparison between rest and stress/exercise conditions. Typically, the relevant metric is obtained via non-invasive imaging (most commonly echocardiography) or via invasive RHC (73). An important aspect of the PAPs linear model is that the concept of ventricular reserve naturally emerges from the resting data. The hydraulic pulmonary pulsatile pressure component is generated by summation of sine-transformed variables and thus varies continuously from a net negative value at normal PAPs to a net positive value at high PAPs. Thus, for a continuous variable such as EF, the sine transform indicates that a high EF value is not necessarily “better” than a low EF value since the sine transform systematically cycles between positive and negative values several times over the expected EF range. Figure 8 illustrates the result of combining several sine-transformed variables to generate the net hydraulic pulmonary pulsatile pressure that trends from negative through zero to positive values as PAPs increases. When all sine waves contribute at maximum positive value, the hydraulic pulmonary pulsatile pressure component cannot increase further. This is represented by the punctuated red line in Figure 8. In contrast, note the result of combining the absolute magnitude of each sine-transformed variable in Figure 8 where it can be appreciated that the absolute magnitudes of each pulsatile pressure variables are not in general different between low and high PAPs levels. That is, the absolute magnitude of each contribution to pulsatile pressure is not the dominant determination of the experienced pressure, instead it is the phase (controlled by the sine transform) of each variable’s contribution to pressure that primarily determines the experienced pressure. Here we define the right ventricular contractile reserve as the net difference between the instantaneous hydraulic pulmonary pulsatile value and the upper limit of 56 mm Hg. Thus, the hydraulic pulmonary pulsatile reserve can vary from a maximum of 112 when the phase of each variable component is wholly negative to 0 when the phase of each variable component is wholly positive. For our PAH cohort the highest hydraulic pulmonary pulsatile reserve was 84 and the lowest was 30. This range is similar to that observed for a study assessing PAPs increase between rest and exercise using echocardiographic estimation, where the 95% confidence limits of PAPs increase were 83 and 13 mm Hg (17).

Of course without a true assessment of cardiac reserve we cannot be certain that the assignment of RV contractile reserve was correct. However, the 6MWD measured in PAH patients is known to be related to impaired oxygen delivery as a result of decreased cardiac index and decreased RV contractile reserve (17, 41). A multi-variable linear model of 6MWD was constructed with the biomechanical elements of height and weight and the right-sided components of hydraulic pulmonary pulsatile pressure and RV impedance match index. Table 4 shows that patient height related to the 6MWD through a sine transform as did the hydraulic pulmonary pulsatile pressure and RV impedance match index. The sine transform of height is likely related to the pendulum effect of each leg taking longer to “swing” for taller people, but accomplishing more distance per step (74). The full model *r*^2^ was 0.45. An intriguing feature is that hydraulic pulmonary pulsatile pressure, which is the result of combining seven sine-transformed variables, was in turn predictive of 6MWD via the sine transform. This provides further evidence that the sine transform is fundamental to the relationship between cardiovascular variables and measures of outcome (such as between 6MWD and pressure components).

### 4.5. Individualized monitoring and management

The components of the linear model of pulmonary pressure were obtained from cardiac-measured variables. This is significant since it has been established that cardiac status, and in particular right ventricular output reserve, is a major determinant of long-term outcome (75). The survival half-life after initial PAH diagnosis is about 5–7 years (76) and assessment via the REVEAL score can further stratify risk (77). However, irrespective of their risk strata, some PAH patients may appear to being doing well and, without any predictors of impending decline, may rapidly deteriorate and die (7). In part this may be a consequence of stratifying patients by an outcomes-based risk factor which does not necessarily group them by common underlying physiology (78). Thus, despite appropriate risk stratification and management at specialized centers of excellence, individual PAH patients remain difficult to assess. The majority of monitoring approaches involve assessment of PA pressure, with the RHC being regarded as the gold standard (79) while some patients are assessed on a daily basis via indwelling pressure monitoring devices (80). However, improvement in pulmonary hemodynamics and quality of life rarely translate to a survival benefit (81). Consequently, use of hemodynamic markers as surrogate end points for trials has declined over time while measures of functional status have increased (82, 83). More recently, trials that rely on time to clinical worsening have been shown to demonstrate survival benefit (84). However, the population size and duration of event-driven trials are dominated by the expected number of events (85) which typically leads to large trials of long duration. In an attempt to reduce trial size and duration it has been proposed that trial entry criteria could be skewed to predominantly include high-risk patients (27). However, this may present a further impediment to trial efficiency in that the therapy has to demonstrate an outcome benefit in patients with the most advanced disease. In traditional trial designs, which include a wide range of patients, it is possible that to a large extent the power of the trial is attributable to the clinical benefit realized by less sick patients. Thus, the ideal trial endpoint should reflect and quantify the degree of clinical benefit in addition to clinical worsening, irrespective of patient symptoms. By this means the difference between trial groups is essentially doubled compared to trials that measure only clinical worsening. The linear model presented here not only offers a means to non-invasively assess PA pressures via measurement of key cardiac variables, but importantly, offers the simultaneous ability to assess cardiac function. This is due to the key cardiac variables (state variables) being intimately involved in characterizing cardiac morphologic and functional status. Thus, the value of the linear model to future clinical trials is that the outcome of the trial could be assessed in terms of a vector of key variables that describe hemodynamic change, cardiac efficiency and RV contractile reserve, which can quantitatively express both beneficial and deleterious changes. The vector of end-points has the added power that even the small changes in several components may be beneficial and that this benefit can be captured and related to hemodynamic and cardiac functional status simultaneously.

Since the linear models provide the infrastructure to directly assess pressure and cardiac status at the component level, they may allow translation of therapeutic benefit between PH and PAH trials. Outside of a randomized clinical trial, assessment of the effects of therapy in PAH patients is hampered by the need to continuously adjust medications in response to evolving symptoms (86). Further, even within a randomized clinical trial, the rarity of PAH patients often renders the trial non-conclusive due to low numbers of trial participants and to the lack of suitable outcomes over the short term (26). Nevertheless, progress has been made in managing PAH patients, while advances in PH are rare. This has led to the unfortunate trend whereby PAH medications are indiscriminately offered off-label to PH patients, often with poor outcomes (87). Given the different etiologies of PH vs. PAH, translation of therapies between PH and PAH populations is fraught with difficulties. In part, these difficulties are the result of an insufficient framework with which to assess patients. The linear models presented here are an attempt to provide a uniform framework with which to assess PAH and PH patients based on hydraulic mean and hydraulic pulsatile pressure components. We note that these two components cannot typically be assessed with a routine RHC evaluation due to the requirement for high-fidelity pressure and flow measuring catheters (88). Further, a given therapy is likely to affect only a few of the variables influencing hydraulic pulmonary mean and pulsatile components, and potentially, while it may improve some components, it may adversely affect others, thus attenuating or even negating any benefit. This mixture of therapeutic benefit and detriment has potential to explain the common observation that some PAH patients improve while others decline, presumably due to differences in the combination of cardiac variables between seemingly similar patients in terms of cardiac status and pressure conditions. Since the linear model components are obtained from non-invasive CMR imaging at rest there is great potential to identify the detailed response of each therapy in a wide-ranging population. This has potential to address an often encountered complication of PAH, namely the deleterious influence on left-sided pressure (89). Since the models simultaneously identify variables that affect right and left pressure conditions there is the possibility that therapeutic interventions can be tailored to maximize the benefit-to-risk ratio on an individual basis.

It is unlikely that any one therapy can provide the required degree of benefit for effective PAH management, instead it may be more desirable to consider combining several therapies in a designer manner (90). While desirable, the feasibility of this approach has been hampered by several factors, including limited knowledge concerning therapeutic interaction and the perceived need to achieve large changes in order to make a clinical difference. Assessment of each trial patient with the linear models may address both of these obstacles. Firstly, the linear models have identified that the contributing variables are independent predictors and that they affect both pulmonary and systemic pressure, thus they each contribute non-overlapping data. Secondly, the linear models indicate that even small changes in key variable can be effective. For example, consider the contribution of EF, if pressure were linearly related to EF then clinically-significant benefit may only be achieved with a dramatic increase in EF. However, the task of raising a patient’s EF from 40 to 60 for example is typically not within the range of effectiveness of any given therapy and the requirement to do so would seem to put the adjustment out of reach. However, here we show that EF is related to pressure via a sine transform. The frequency of sinusoidal oscillation over the EF axis is on the order of a complete cycle every 10 EF points. Thus, in this case, adjustments on the order of 5 EF points may be all that are required to beneficially change the phase of a pressure contribution. With reference to Figure 8 we can appreciate that the magnitude of the combined modeled variables do not dramatically change over the PAPs range, whereas the phase changes of each individual component dominate the resultant PAPs and RV reserve. Thus, achieving a beneficial effect may be more feasible than previously thought.

### 4.6. Limitations

The study has a number of strengths and also many deficits and limitations, most notably the lack of measured PAPs in PH and some PAH patients, the lack of a stress-test assessed measure of RV pulsatile reserve, the lack of a separate model generation and model assessment population and the use of clinically-obtained SBP. The PAH patients from the Complexa trial were well documented, but only a minority of the retrospectively obtained patients undergoing a functional CMR examination had an estimate of PAPs. However, assuming that data were missing at random, the distribution of estimated PAPs was sufficient to confirm the suspicion of elevated PAPs. Further, we lacked a normal PAPs population for model testing. The excellent correlation coefficient *r*^2^ (0.89) for the model agreement with PAPs is gratifying, but as Bland and Altman indicate, a good correlation is necessary but not sufficient to warrant substitution of one test for another (91). The corresponding Bland-Altman analysis in Figure 1 shows that the 95% limits of agreement are ±11 mm Hg. These limits, while not ideal, are lower than is traditionally achieved with echocardiography which are on the order of ±28 mm Hg (92). While we did not have sufficient data for test and prediction sub-sets, an estimate of the robustness of the model was obtained via bootstrapping, which indicated an excellent mean correlation *r*^2^ of 0.92 with a standard deviation of 0.04 on the estimate of PAPs. The accuracy of model components can be estimated by comparing the modeled SBP with the directly measured SBP, but it is unknown to what extent the model may be adequate to screen for PH. Our assignment of component combinations to hydraulic pulsatile and hydraulic mean pressure was not confirmed by any direct measure of the hydraulic power distribution. The magnitudes of hydraulic pulmonary pulsatile and hydraulic systemic pulsatile are about one quarter of the magnitudes of hydraulic pulmonary mean and hydraulic systemic mean pressure when the constant offsets are assigned to the mean pressure components. This agrees with the observation that typically the oscillatory hydraulic power is about 30%–40% of the total hydraulic power (93). Our motivation for seeking a sinusoidal transform to relate cardiac features to the pressure data originated with an observation by the pioneers of cardiac energetics, Suga and Sagawa. Sinusoidal transforms allowed excellent fit of the data. The frequencies identified were not uniform, which in part is attributable to the different units for each variable and in part likely due to sensitivity to the fundamental as well as harmonic frequencies which are known to be present (94). Development of a schema that relates the sinusoidal transforms to a common axis might further add to the development of the model and further support its conceptual basis. Figure 3A shows that hydraulic pulmonary mean and hydraulic systemic mean pressures are almost perfectly antisymmetric. It might be thought that this is an artifact or natural consequence of the model. However, this is not the case since the relationship is not perfectly antisymmetric due to the differences in model coefficients between PAPs and SBP (Table 3): coefficients for age, RV mass and LV mass for SBP/PAPs are 0.45/−0.68, −0.26/0.39 and 0.17/0, respectively. Whether this relationship should be exactly antisymmetric is not known, but if it were, then adding this constraint to the model would further refine and improve it. One of the most gratifying aspects of the model is that it naturally suggests an upper limit for the RV and LV contractile function, i.e., contractile reserve. Indirect evidence that our identification of RV contractile reserve is related to the true reserve is that it significantly contributed to the 6MWD model (95). Further, clinical approaches to asses RV contractile reserve themselves have assumptions, and isolating which aspects relate specifically to RV contraction might be difficult to establish (96).

### 4.7. Summary of findings

In conclusion we have presented a pair of unified models of PAPs and SBP in PAH and PH patients with physiologically feasible assignment of components to hydraulic mean and hydraulic pulsatile pressures. The model indicated that both right and left ventricular variables contributed to right and left pressure either linearly or via sine transforms. It is possible that model refinements could be identified, such as incorporating a transform derived directly from time-resolved cardiac data. However, the fundamental connection between right and left pressure generation is expected to persist. Beyond modeling PAPs and SBP, successes of the model include natural emergence of the concept of RV contractile reserve which was shown to correlate with the 6MWD, the decrease in systemic perfusion pressure in advanced PAH patients, and the progressively poor performance of the LV in PAH patients despite a trend to increase LV mass. These features are consistent with a large body of clinical observations. Of primary importance, the model identified a very distinctive pattern of pulmonary pulsatile pressure that was different between PAH and PH patients. The pulsatile patterns were physiologically feasible given the pre vs. post capillary constrictions in PAH vs. PH, respectively. We regard the models as providing a universal framework with which to assess the results of therapy applied to PAH and PH patients.

## Data Availability

The raw data supporting the conclusions of this article will be made available by the authors, without undue reservation.
