# Supplementary material for: The sine transform is the sine qua non of the pulmonary and systemic pressure relationship
Source: Front Cardiovasc Med. 2023 May 26;10:1120330. doi: 10.3389/fcvm.2023.1120330 (PMC10250723; doi:10.3389/fcvm.2023.1120330)
Supplement: Supplementary file 1 [file Table1.docx]

# Supplemental material

## Table: Performance sites and local PIs of the Complexa Trial.

| Local PI | Institution |
| --- | --- |
| Raymond Benza, MD | Allegheny Health Network, Pittsburgh, PA |
| Franz Rischard, DO | Banner University Medical Center, Tucson, AZ |
| Gustavo Heresi, MD | Cleveland Clinic, Cleveland, OH |
| Kishan S. Parikh, MD | Duke University Medical Center, Durham, NC |
| A Colin Church, MD | Golden Jubilee National Hospital, Clydebank, UK |
| Zeenat Safdar, MD | Houston Methodist, Houston, TX |
| Luke Howard, MD | Imperial College Healthcare NHS Trust, Hammersmith Hospital, London, UK |
| Oksana Shlobin, MD | Inova Fairfax MRI Center, Falls Church, VA |
| Tim Lahm, MD | IU Health Methodist Hospital MPC, Indianapolis, IN |
| M. Patricia George, MD | National Jewish Health, Denver, CO |
| Roxana Sulica, MD | New York University, New York, NY |
| Gerry Coghlan MD | Royal Free, London, UK |
| Michael Eggert, MD | Sentara Norfolk General Hospital, Norfolk, VA |
| Jeremy Feldman, MD | St. Joseph’s Hospital and Medical Center, Phoenix, AZ |
| Peter Engel, MD | The Christ Hospital, Cincinnati, OH |
| Jimmy Shaun Smith , DO | The Ohio State University Preoperative Assessment Center, Columbus, OH |
| Leslie A Spikes, MD | The University of Kansas Hospital, Kansas City, KS |
| Joana Preston, MD | Tufts Medical Center, Boston, MA |
| Hernando Garcia, MD | University Medical Center of El Paso, El Paso, TX |
| Mandar Aras, MD | University of California SFO, San Francisco, CA |
| Remzi Bag, MD | University of Chicago Medicine, Chicago, IL |
| Kurt Prins, MD PHD | University of Minnesota, Minneapolis, MN |
| Melendres-Grove, MD | University of New Mexico, Albuquerque, NM |
| Jeremy Mazurek, MD | University of Pennsylvania, Philadelphia, PA |
| Andrew Mihalek, MD | University of Virginia Health System, Charlottesville, VA |
| Sonja Bartolome, MD | UT Southwestern Medical Center, Dallas, TX |
| Ivan Robbins, MD | Vanderbilt University Medical Center, Nashville, TN |
| Murali Chakinala, MD | Washington University School of Medicine, St. Louis, Missouri, MO |
